# Gender and socioeconomic disparities in BMI trajectories in the Seychelles: a cohort analysis based on serial population-based surveys

**DOI:** 10.1186/1471-2458-11-912

**Published:** 2011-12-09

**Authors:** Isabelle A Rossi, Valentin Rousson, Bharathi Viswanathan, Pascal Bovet

**Affiliations:** 1 Institute of Social and Preventive Medicine (IUMSP), Lausanne University Hospital, Lausanne, Switzerland; 2 NCD Section, Department of Public Health, Ministry of Health, Victoria, Republic of Seychelles

## Abstract

**Background:**

The relationship between body mass index (BMI) and socioeconomic status (SES) tends to change over time and across populations. In this study, we examined, separately in men and women, whether the association between BMI and SES changed over successive birth cohorts in the Seychelles (Indian Ocean, African region).

**Methods:**

We used data from all participants in three surveys conducted in 1989, 1994 and 2004 in independent random samples of the population aged 25-64 years in the Seychelles (N = 3'403). We used linear regression to model mean BMI according to age, cohort, SES and smoking status, allowing for a quadratic term for age to account for a curvilinear relation between BMI and age and interactions between SES and age and between SES and cohorts to test whether the relation between SES and BMI changed across subsequent cohorts. All analyses were performed separately in men and women.

**Results:**

BMI increased with age in all birth cohorts. BMI was lower in men of low SES than high SES but was higher in women of low SES than high SES. In all SES categories, BMI increased over successive cohorts (1.24 kg/m^2 ^in men and 1.51 kg/m^2 ^for a 10-year increase in birth cohorts, *p *< 0.001). The difference in BMI between men or women of high vs. low SES did not change significantly across successive cohorts (the interaction between SES and year of birth of cohort was statistically not significant). Smoking was associated with lower BMI in men and women (respectively -1.55 kg/m^2 ^and 2.46 kg/m^2^, *p *< 0.001).

**Conclusions:**

Although large differences exist between men and women, social patterning of BMI did not change significantly over successive cohorts in this population of a middle-income country in the African region.

## Background

The prevalence of overweight and obesity has increased during the few past decades in populations of high- and middle-income countries as well as in low-income countries [1-3]. Obesity tends to be strongly associated with gender and socioeconomic status (SES) but the direction of these associations varies according to the levels of economic development [4-8].

Obesity has been associated with lower SES in both genders in developed countries [8] but with high SES in several developing countries, at least until the late 1980s [4,5,9]. However, a shift of obesity from higher to lower SES groups is increasingly observed in developing countries as a country's GDP increases, as it has been demonstrated in reviews of single cross-sectional studies [4,9] as well as in repeated cross-sectional studies [10]. Consistent with these observations, a recent review in 37 developing countries showed that obesity occurred at a faster pace among persons of lower SES in countries experiencing economic development [11]. Studies on the relationship between SES and obesity have often been limited to women only [9,11-13] and few provide analyses of trends over time [10,11,13,14]. There is a need for further research focusing on trends in obesity according to SES [15].

In contrast with findings in several developing countries in Africa [13,16-18] and other regions showing direct associations between obesity and high SES in both men and women [4,11,19], data in the Seychelles, a rapidly developing country in the African region, show that obesity is associated with lower SES among women, but with higher SES in men [14].

However, most of the findings on trends in social patterning rely on a comparison of data from successive cross-sectional surveys while only a few studies were based on longitudinal data [20-22] and none of them were performed in the African region. When comparing data from consecutive cross-sectional surveys, one is actually estimating a period effect rather than a cohort effect (note that we are using throughout this paper the commonly used term of "effect" without an interpretation of causality). Yet, such an analysis cannot adequately describe the weight gain over the life course of individuals and hence does not allow assessing whether the associations between BMI, age and SES categories change over successive cohorts (i.e. among persons born at different times).

Hence, using data from three independent population-based surveys conducted over a 15-year period in the Seychelles, we modeled mean BMI according to age, SES and year of birth (i.e. the "cohort effect") separately in men and women. The aim of this study was to examine whether the association between BMI and SES had changed over successive birth cohorts of men and women in the Seychelles.

## Methods

The Republic of Seychelles consists of over 100 islands located in the Indian Ocean, about 1800 km east of Kenya, in the African region. Around 90% of the population lives on the main island and the majority of people are of African descent. The GDP per capita has increased, in real values, from 2927 $ in 1980 to 5239 $ in 2004, driven by booming tourism, industrial fishing and services [23]. The Seychelles can be considered as urbanized or semi-urbanized in view of a high population density, the fact that a large proportion of the population regularly commutes to town for work, and the increasing role of services in the overall economy, in addition to tourism and fishing industries [24].

Three independent population-based surveys of cardiovascular risk factors were conducted in independent representative samples of all adults aged 25-64 years in the Seychelles. These population-based surveys were performed in 1989, 1994 and 2004, respectively. All surveys were approved by the Ministry of Health after technical and ethical reviews. Participants were free to participate and gave informed consent. The sampling frame, methods and main results of the three surveys have been described previously [25-28]. Briefly, the sampling frame of each survey consisted of an age- and sex-stratified random sample of the total population aged 25-64 years. Eligible participants were selected from an electronic database derived from population censuses, thereafter regularly updated by civil status authorities. The surveys were attended by 1081 persons in 1989 (86.4% participation rate), 1067 in 1994 (87%), and 1255 in 2004 (80.2%). Total numbers of participants for the three surveys comprised 1585 men and 1818 women. It can be expected that only around 30 persons have participated by chance in two surveys while only 1-2 persons have participated in all 3 surveys. There were no missing data for the considered variables.

Height was measured at 1 cm precision and weight was measured at 0.1 kg precision by trained survey officers using standard and validated weighing scales and stadiometers. BMI was calculated as weight (kg) divided by height (m) squared. Smoking status was assessed from a questionnaire. In all three surveys, the same question classified occupation in six categories, based on the participant's current occupation or his/her past occupation if a participant was not currently employed. The classification of occupation along six classes ranked from higher to lower prestige or social standing is consistent with the British occupation-based Registrar' Social Classes [29]. In this paper, we grouped the 6 categories into three categories. The highest category included "professionals" and "skilled non manuals", the intermediate category included "semi skilled manual", "skilled manuals", "and semi skilled non-manuals" and the lowest category included "unskilled workers" [14].

Data collected from successive cross-sectional surveys can be modeled either in terms of age and cohort effect or in terms of age and period effect. We chose to present results in terms of age and cohort effect. Thus, separately for men and women, we considered models for BMI including a linear effect and a quadratic effect of age (to allow for curvilinear relationship between BMI and age) and a linear effect of cohort (i.e. the year on which a person is born). We included an interaction term between SES and age and an interaction term between SES and cohort (year of birth) to test whether the relation between SES and BMI varied by age, respectively by cohort. The models were also adjusted for smoking. Of note, a quadratic effect for cohort (year of birth) was not significant (*p *= 0.56 for men and *p *= 0.77 for women for the model used in Table 1). We have also tried to replace the linear effect of cohort by a categorical effect of cohort with five possible values (year of birth < 1935, 1935-1944, 1945-1954, 1955-1964, after 1965). This did not result in a better fit. For example, the adjusted R^2 ^values were slightly smaller using a categorical cohort effect vs. using a linear effect. Thus, the linear regression model that we use in this paper was a parsimonious and convenient approximation of the reality and it is useful for testing statistically our hypothesis that social patterning of BMI had changed over time.

**Table 1:** Regression coefficients of linear regression model to explain BMI according to age, birth cohort, socio economic status, and smoking status, separately for men and women

	Men (n = 1585, adj. R^2 ^= 0.12)		Women (n = 1818; adj. R^2 ^= 0.11)	
	
	Coefficient	*P*	Coefficient	*P*
Intercept*	23.61		27.99	

Current smoking	-1.55	< 0.001	-2.46	< 0.001

Middle SES (vs. low)	1.46	< 0.001	-0.88	0.004

High SES (vs. low)	1.69	< 0.001	-2.09	< 0.001

Age (10 years)	1.65	< 0.001	2.57	< 0.001

Age^2 (10 years)	-0.32	< 0.001	-0.56	< 0.001

Cohort (10 years)	1.24	< 0.001	1.51	< 0.001

*The intercept refers to a non-smoking person aged 45 born in 1944 and with a low SES.

All models and tests, as well as the corresponding figures, were performed using the R (version 2.5.1) free statistical package. Two tailed p values < 0.05 were considered significant.

## Results

The distribution of the occupation categories did not vary substantially according to gender (Table 2). However, the prevalence of the "professional" category increased over successive surveys in both genders, especially between 1989 and 1994. In each survey, mean BMI and the prevalence of overweight (BMI ≥25 kg/m^2^) and obesity (BMI ≥30 kg/m^2^) were higher in women than in men. All adiposity markers increased significantly in both genders between 1989 and 2004.

**Table 2:** Distribution of occupation and body mass index categories according to sex and survey year

		Men			Women		
		
		1989 (513)	1994 (504)	2004 (568)	1989 (568)	1994 (563)	2004 (687)
Occupation							

Low SES	%	39.0 (2.1)	30.0 (2.0)	28.2 (1.9)	53.5 (2.1)	38.2 (2.0)	49.5 (1.9)

Middle SES	%	52.1 (2.2)	54.8 (2.2)	56.5 (2.1)	34.9 (2.0)	44.9 (2.1)	33.0 (1.8)

High SES	%	9.0 (1.3)	15.3 (1.6)	15.3 (1.5)	11.6 (1.3)	16.9 (1.6)	17.5 (1.4)

Body mass index							

Mean BMI (kg/m^2^)		23.3 (0.2)	24.1 (0.2)	25.5 (0.2)	25.9 (0.2)	26.9 (0.3)	28.3 (0.2)

BMI ≥25 (kg/m^2^)	%	28.1 (2.0)	38.7 (2.2)	53.2 (2.1)	55.3 (2.1)	61.1 (2.1)	71.3 (1.7)

BMI ≥30 (kg/m^2^)	%	4.5 (0.9)	8.5 (1.2)	15.9 (1.5)	25.2 (1.8)	29.1 (1.9)	37.7 (1.9)

Results are presented as mean or percentage, with standard error between brackets

*SES *socio economic status

**Figure 1:**
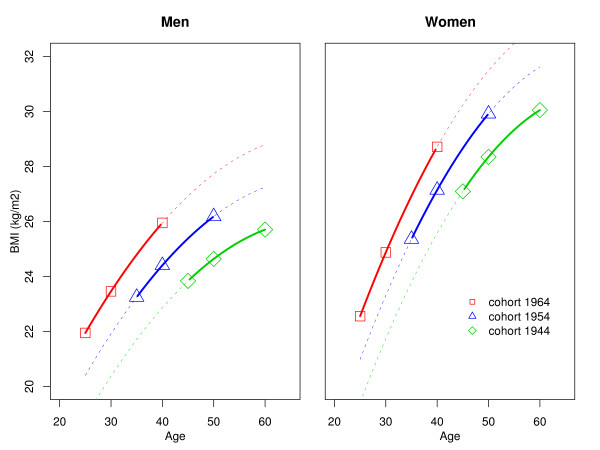
**BMI trajectories according to age and birth cohort in men and women, as predicted by linear regression**. Models include a quadratic term for age and are not adjusted for other covariates. Years of birth for the cohorts displayed (1944, 1954, 1964) are arbitrary but maximize the ranges of participants' ages during which predicted BMI values are based on data estimated from the underlying surveys (plain lines) vs. extrapolated predicted values (dotted lines).

Figure 1 shows the fit of a model relating BMI according to age and birth cohorts while Table 1 shows the results of the statistical models relating BMI to age, birth date (i.e. cohort), SES and smoking, separately for men and women. There is a marked cohort effect, with an estimated BMI increase of 1.24 and 1.51 kg/m^2 ^for each 10-year increase for men and women, respectively. Both a middle SES and high SES are significantly associated with a higher BMI for men and with a lower BMI for women.

**Figure 2:**
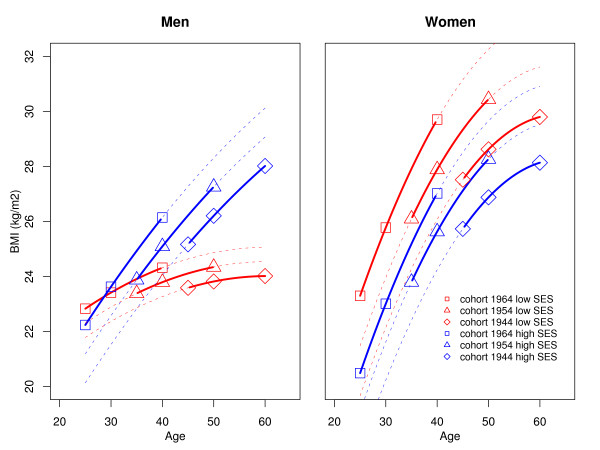
**BMI trajectories according to age, birth cohort and socio-economic status category in men and women, as predicted by linear regression**. Models are also adjusted for smoking status and include a quadratic term for age and interaction terms between SES and age and between SES and cohort (year of birth)

Table 3 shows the results of a model where interactions between the cohort effect and the SES effect and between the age effect and the SES effect are introduced. The corresponding fits are plotted in Figure 2. Since the BMI trajectory of the intermediate occupation category was intermediate as compared to the BMI trajectories for the low and high SES categories, and for better readability of the figures, we excluded persons with intermediate SES from analyses displayed in Table 3 and in Figure 2 (hence only the "high" and "low" SES categories are displayed). Table 3 shows that there is no significant SES-cohort effect, which suggests no change in social patterning of BMI over time.

**Table 3:** Regression coefficients of linear regression model to explain BMI according to age, birth cohort, socio-economic status, and smoking status, separately for men and women, with an interaction of SES with age and an interaction of SES with cohort

	Men (n = 716, adj. R^2 ^= 0.14)		Women (N = 1140; adj. R^2 ^= 0.11)	
	
	Coefficient	*P*	Coefficient	*P*
Intercept*	23.91		28.00	

Current smoking	-1.46	*P *< 0.001	-2.17	*P *= 0.001

High SES (vs. low)	1.58	*P *= 0.003	-1.79	*P *= 0.011

Age (10 years)	0.55	*P *= 0.073	2.55	*P *< 0.001

Age^2 (10 years)	-0.18	*P *= 0.145	-0.69	*P *< 0.001

Cohort (10 years)	0.52	*P *= 0.050	1.82	*P *< 0.001

Age * high SES	1.61	*P *= 0.004	0.08	*P *= 0.908

Cohort * high SES	0.53	*P *= 0.294	-0.43	*P *= 0.514

*The intercept refers to a non-smoking person aged 45 born in 1944 and with a low SES. Persons of intermediate SES are omitted from these analyses

With regards to the interaction between SES and age, it was significant in men, but not in women. The significant interaction between the age effect and the SES effect in men is clearly visible on the left panel of Figure 2: the difference in BMI between low and high SES (with a higher BMI for persons with high SES and a lower BMI for persons with low SES) is increasing with age in all cohorts. An interaction between age and SES is not present in women: the difference of BMI between low and high SES (with lower BMI for the latter) remained sTable across age. Smoking was significantly associated with a lower BMI in all models.

## Discussion

To our knowledge, this is the first study in the African region to attempt to disentangle the distinct effects of age and birth cohort when studying the evolution of the relationship between SES and BMI. Firstly, BMI increased over time across successive cohorts of men and women. Secondly, BMI was lower in men of low than high SES but higher in women of low than high SES. Thirdly, and this was the main aim of this study, the social patterning of BMI did not change markedly across successive birth cohorts (which can also be interpreted as a lack of change in social patterning of BMI over time).

Studies showing secular trends in obesity in African populations [11-14] were based on the comparison of findings from successive surveys, an analysis that focuses on the "period effect", and these studies showed that BMI increased among participants across successive surveys. Ideally, one would like to be able to separate the distinct effects of age, period and cohort, which is however not possible due to their colinearity. When comparing data from repeated cross-sectional surveys, one should decide a priori whether to estimate a period effect or a cohort effect. In this study, we modeled BMI in relation to age, birth cohort and SES (hence allowing examining the "cohort effect", i.e. the weight gain over the life course of individuals across successive birth cohorts). We found a marked cohort effect, i.e. BMI increased by more than 1 kg/m^2 ^per increase of 10 years of birth date of successive cohorts. Hence, newer generations had a higher BMI than previous generations at a same age. It is useful to note that the observed increase of BMI according to participants' age (e.g. when plotting the relationship between BMI and age using data of a single cross sectional survey) will markedly underestimate the true effect of age on BMI at a cohort level (i.e. when looking at the increase of BMI according to age among persons born on same years). Indeed older persons in subsequent surveys come from older cohorts who had lower BMI at a same age. More generally, a gradually higher BMI at a same age in successive cohorts (i.e. a linear effect of cohort, as found in this study) is consistent with a period effect (i.e. a higher BMI among all participants across successive surveys, as previously shown with the same data [14]).

An inverse association between BMI and SES in women is often found in developed countries [6-8,21] and has been reported in an increasing number of middle-income developing countries [4,8,10,30], including in the Seychelles [14]. An association between BMI and SES is less consistent in men, at least in developed countries [6-8]. In developing countries, obesity tends to shift from higher to lower SES groups as the country's GDP increases [4,8,11,30]. This shift generally occurs at an earlier stage of socioeconomic development in women than in men [4]. In the Seychelles, we found that a higher BMI was associated with high SES in men but with low SES among women.

The main finding of this study is that social patterning of BMI did not change markedly across consecutive cohorts. Increasing BMI in all SES groups suggests exposure to common environmental obesogenic factors [9,13,31,32]. Rapid socioeconomic development may be associated with increased food intake and sedentary lifestyle in the Seychelles, which may have occurred similarly in all SES categories during the past two decades. Factors commonly associated with lower BMI in lower SES groups in developing countries, such as food scarcity and/or high energy expenditure activities, may no longer be major factors underlying trends in BMI in the population in the Seychelles. Seychelles has been experiencing a fairly high social welfare since three decades and high employment rates among both men and women in all SES categories. This context may underlie the lack of substantial change in social patterning over time in Seychelles. However, from cultural and social perspectives, it has been suggested that men in developing countries value a large body size (which may be viewed as a sign of physical dominance and prowess), while women increasingly value a leaner weight, perhaps in order to emulate western leanness standards [8,33]. Consistent with these trends, recent studies on self perception of body weight among adults and children in Seychelles showed greater acceptance of high BMI in males than females [34] and in persons of low than high SES [35]. Further qualitative studies would be useful to help clarify these potentially important factors and their relationship with weight, according to gender and birth cohorts.

Limitations of the study include a fairly short follow-up for each birth cohort (15 years). Furthermore, SES in our study relied on one single occupation indicator [36]. However, occupation is a fair indicator of SES if the employment rate is high, while education may be a less reliable indicator in developing countries in view of major changes in education systems over the past decades [37]. In the Seychelles, employment rate was > 90% in 2004. With regards to education, 86% of persons aged 25-34 years in 2004 had completed secondary school vs. only 11% of persons aged 55-64 years in 1989 [14]. Strengths of this study include the availability of three population-based surveys over a fifteen-year period, the availability of measured BMI data (i.e. not self-reported), and the classification of SES based on the same questionnaire in all surveys.

## Conclusion

Based on a cohort analysis using regression analysis of data from three population surveys in 1989, 1994 and 2004 in the Seychelles, we found marked differences in social patterning of BMI in men and women and that BMI increased markedly over successive cohorts within all SES categories, but the relationship between BMI and SES did not change significantly over time. It will be interesting to examine this question again when data from next population-based surveys become available. This study also illustrates that it is possible, using data from serial population surveys, to use cohort analysis to disentangle the distinct effects of age and birth cohort when studying the evolution over time of the relationship between SES and BMI.

## Competing interests

The authors declare that they have no competing interests.

## Authors' contributions

IAR led the analysis of data and the write up of the manuscript. VR performed the statistical models and participated in the write up of the manuscript. BW participated in the surveys and reviewed the manuscript. PB was the PI of the three surveys and participated in data analysis and in the write up of the manuscript. All authors reviewed and approved the final manuscript.

## Pre-publication history

The pre-publication history for this paper can be accessed here:

http://www.biomedcentral.com/1471-2458/11/912/prepub
